# Psychometric properties of the French borderline symptom list, short form (BSL-23)

**DOI:** 10.1186/s40479-016-0038-0

**Published:** 2016-06-10

**Authors:** Rosetta Nicastro, Paco Prada, Anne-Lise Kung, Virginie Salamin, Alexandre Dayer, Jean-Michel Aubry, Florence Guenot, Nader Perroud

**Affiliations:** Service of Psychiatric Specialties, Department of mental Health and Psychiatry, University Hospitals of Geneva, 20bis rue de Lausanne, 1201 Geneva, Switzerland; Fribourg Mental Health Network, Fribourg, Switzerland; Department of Psychiatry, University of Geneva, Geneva, Switzerland

**Keywords:** Borderline personality disorder, Dialectical Behavior Therapy, Self-report questionnaire, Emotional lability, Impulsivity

## Abstract

**Background:**

The short form of the Borderline Symptom List (BSL-23) is a self-rating instrument used to assess specific symptoms of borderline personality disorder (BPD). The original German version has shown good psychometric proprieties. The BSL-23 can also be used to measure the effects of therapy on patients with BPD. The aim of this study was to assess the psychometric properties of the French version of the BSL-23.

**Methods:**

The French version of the BSL-23 was given to 265 subjects with BPD. Factor structure, reliability, test-retest stability, convergent validity, divergent validity, and sensitivity to change were analysed. Forty-five subjects suffering from attention-deficit hyperactivity disorder (ADHD) were used as controls to evaluate the specificity of BSL-23.

**Results:**

A one-factor structure was obtained in the French version of the BSL-23, showing high internal consistency (Cronbach’s alpha = .94) and test-retest reliability (*r =* .841). The French version of the BSL-23 was highly correlated with depression severity, hopelessness, anger, motor impulsiveness, and BPD diagnosis. It was an efficient tool to discriminate between BPD patients and ADHD patients, and showed good sensitivity to change in a group of BPD patients who took part in a one-month DBT intervention.

**Conclusions:**

The French version of the BSL-23 shows similar psychometric properties as the original German version. This study therefore provides clinicians and researchers with a French instrument to measure BPD symptomatology.

## Background

Borderline personality disorder (BPD) is characterized by emotional dysregulation, impulsivity, self-damaging and suicidal behaviours, interpersonal difficulties, and identity disturbance. The lifetime prevalence of BPD among the general population varies according to surveys, diagnostic instruments and rules, but it is estimated to be in between 0.7 % and 2.7 % in recent studies [1–3]. BPD is a severe condition that causes major impairments in a variety of contexts and is associated with poor socio-economic and familial outcomes [4]. BPD patients are frequent users of mental health services and their mortality rate by suicide reaches 10 % [5]. The relevance of early diagnosis has been demonstrated by Kaess et al. [6], who showed that BPD can be identified during adolescence.

In order to establish a diagnosis for BPD based on the DSM-5 criteria [7], clinicians and researchers commonly use structured interviews, such as the Structured Clinical Interview for DSM-5 Axis II Personality Disorders (SCID-II) [8]. In addition to the interviews, clinician-rated scales, such as the Zanarini Rating Scale for Borderline Personality Disorder [9] and the Borderline Personality Disorder Severity Index [10], are used as screening tools for BPD symptomatology or as instruments assessing changes in the severity of the disorder. Finally, self-report scales have been developed to take into account the subjective view patients have of their disorder. These scales include, but are not limited to, the Borderline Evaluation of Severity over Time [11] and the Borderline Symptom List (BSL) [12, 13], both quantifying borderline-specific symptomatology.

The initial BSL [12, 13] included 95 items based on the criteria of the DSM-4, the Diagnostic Interview for Borderlines-Revised (DIB-R) [14], and the opinions of clinical experts and BPD patients. Each item describes a complaint frequently made by BPD patients, such as “*I was lonely*” or “*I experienced stressful inner tension*”. The patients are asked to evaluate the intensity of each complaint over the previous week on a 5-point Likert scale, ranging from 0 (none) to 4 (very strong). The BSL-95 showed good psychometric properties [13]. No particular effect of gender, age or level of education was found. Based on the 95-item scale in German, a shorter, 23-item version of the BSL was developed [15, 16]. The BSL-23 was validated on different samples representing a total of 659 BPD patients. The psychometric properties of the BSL-23 were similar to those of the BSL-95 and the correlation between the two versions of the scale was high (range: 0.958–0.963 in five different samples). The factor analysis showed a one-factor structure, and the internal consistency of the BSL-23, as well as its test-retest reliability, were more than satisfactory (Cronbach’s alpha: 0.935–0.969 and *r =* 0.82; *p <* 0.0001). Furthermore, the BSL-23 discriminated BPD patients from healthy subjects and from patients suffering from other psychiatric disorders. It showed a positive correlation with measures of psychopathology, depression and anxiety, and a negative correlation with a measure of global well-being. Finally, the BSL-23 was sensitive to change [15, 16] after three months of dialectical behavior therapy (DBT) [17]. The BSL-23 was also translated and validated in Spanish. The Spanish BSL-23 [18] replicated the one-factor structure of the original version and was found to be a reliable and valid instrument for assessing BPD severity and sensitivity to change. Moreover, it also correlated with depressive symptomatology, state and trait anxiety, hostility and impulsivity scores.

Only a few validated instruments are available in French to specifically assess the severity of BPD symptomatology, and to our knowledge there are no validated self-report scales currently available. The BSL-23 is a brief, sensitive, easy to use and specific instrument, which can be repeated to assess changes in the severity of the disorder over time. Our aim was to examine the structure and psychometric properties of a French version of the BSL-23 on a sample of BPD patients. Factor structure, internal consistency, and test-retest reliability were assessed. Correlations between the French BSL-23 and other psychiatric symptoms were explored. Furthermore, the instrument’s relevance in discriminating BPD patients from a sample of patients suffering from attention deficit and hyperactive disorder (ADHD) was also examined. Finally, sensitivity to change after a four-week Intensive DBT (I-DBT) [19, 20] was tested on a sample of 92 BPD patients.

## Methods

### Participants

BPD patients were recruited in two specialized outpatient units (Geneva and Fribourg) treating patients suffering from BPD and/or ADHD and relying on DBT as a first-line treatment. Patients were interviewed by general practitioners or psychiatrists to assess emotion dysregulation, impulsive behaviours, self-damaging behaviours, and/or suspicion of BPD. Each patient was interviewed first by a trained psychiatrist or psychologist, and then assessed for psychiatric disorders with the Diagnostic Interview for Genetic Studies (DIGS) [21], as part of a broader study investigating genetic and epigenetic correlates of BPD [22]. BPD was assessed with the BPD part of the SCID-II [8]. Only subjects filling the criteria for BPD (5 or more DSM-5 criteria) were included in the study. Psychotic disorder, bipolar affective disorder type 1, and pervasive developmental disorder were used as exclusion criteria. In order to test sensitivity to change of the BSL-23, 92 BPD patients were reassessed after a four-week I-DBT program [19, 20]. I-DBT is an original adaptation of DBT skills training which combines, in a short and intensive format, individual sessions with the primary therapist and skills-training groups based on the traditional DBT modules: mindfulness, interpersonal effectiveness, emotion regulation, and distress tolerance. Patients are also offered telephone assistance with therapists between 9 am and 6 pm. All therapists attend weekly meetings with the consultation team.

Forty-five patients suffering from ADHD were also recruited in order to test the discriminant validity of BSL-23. The ADHD diagnosis was based on a clinical evaluation by a trained psychiatrist and on the Diagnostic Interview for ADHD in adults (DIVA 2.0) [23]. In addition, patients completed the following questionnaires: the Adult ADHD Self-Report Scale (ASRS v1.1) [24], which assesses severity of adult ADHD, and the Wender Utah Rating Scale (WURS) [25, 26], featuring a subset of 25 questions on a five point Likert-scale. Following Fossati et al. [27], we used a very stringent cut-off score of 46 to indicate the existence of ADHD in childhood. BPD was clinically excluded by the same clinicians.

### Assessment

At admission, each patient completed the following self-report scales:

#### The borderline symptom list (BSL-23)

Each subject completed the French version of the BSL-23 to assess BPD symptomatology. BSL-23 [15, 16] was translated from English to French by NP and PP and an independent English-speaking translator back-translated the French version into English. In its original form, the BSL-23 is a 23-item self-rated scale presenting a one-factor structure and high internal consistency (Cronbach’s alpha = .935). The original BSL-23 also boasts good reliability for BPD diagnosis and discriminates BPD patients from other psychiatric patients (mean effect size =1.13). It has also shown a sensitivity to change through therapy.

#### Other self-report measurements

The Beck Depression Inventory II (BDI-II) [28] assesses the current severity of depression symptoms. It includes 21 items that are rated on a four-point scale (0 to 3), with scores ranging from 0 to 63. High scores indicate greater severity.

The Beck Hopelessness Scale (BHS) [29] was used to estimate the degree of pessimism and negativity about the future. Featuring 20 true–false statements, the scores of the scale range from 0 to 20. High scores indicate a greater sense of hopelessness.

The Barrat Impulsivity Scale (BIS-10) [30] is a measure of impulsiveness that includes 34 items rated on a four-point scale (rarely/never, occasionally, often, almost always/always. The scoring of the items reveal three factors: motor impulsivity, cognitive impulsivity, and non-planning impulsivity. High scores indicate a greater level of impulsiveness.

The State-Trait Anger Expression Inventory (STAXI) [31] is a 44-item self-report measure of the experience and expression of anger. Items are rated on a four-point frequency scale and scores range from 0 to 132. Five subscales are calculated: state anger, trait anger, anger-in, anger-out, and anger control, which assesses the intensity of the angry feelings or the frequency at which anger is experienced, expressed, or controlled.

The demographic data (Table 1) were obtained from a standard questionnaire given to all participants. The study was approved by the ethics committees of Fribourg and of the Geneva University Hospital. Patients signed an informed written consent form.

**Table 1:** Clinical characteristics (*N =* 265)

		Mean	SD
Age		32.2	8.9
Education (years)		13.8	2.4
		N	%
Gender	Women	239	90.2
Marital status	Single	186	70.2
	In couple	55	20.8
	Divorced or widowed	24	9.1
Children	0	194	73.2
	1	41	15.5
	2	24	9.1
	3 or more	6	2.3
Activity	Employed or Student	149	56.2
	Unemployed	116	43.8
SCID-II, Number of BPD criteria	5	74	27.9
	6	42	15.8
	7	50	18.9
	8	46	17.4
	9	53	20.0

### Statistical analysis

Data analysis was carried out using SPSS version 22 and STATA release 13. Descriptive statistics were used to describe the demographic and clinical characteristics of the sample. To test internal consistency, a global Cronbach’s alpha was estimated and the split-half method was applied. In addition, Cronbach’s alphas were estimated, with each of the 23 items removed one-by-one from the scale. Test-retest reliability was evaluated by paired-sample correlations.

To measure the appropriateness of the factor analysis, the Kaiser-Meyer-Olkin (KMO) measure of sampling adequacy and the Bartlett’s test of sphericity were used. An exploratory factorial analysis (EFA) of principal components with a Promax rotation was performed to examine the factorial structure of the scale. A confirmatory factor analysis (CFA) was then performed to test the adequacy of the one-factor model proposed by Bohus et al. [16]. The accuracy of the fit with the original version of BSL-23 was tested with chi-squares; as chi-squares are dependent on sample size, other indexes recommended by Hu and Bentler [32] were also used: the standardized root mean square residual (SRMR) and the root mean square error of approximation (RMSEA). Schermelleh-Engel et al. [33] consider that an RMSEA between 0 and .05 indicates a good fit, whereas an RMSEA between .05 and .08 is considered an acceptable fit, and values between .08 and .10 are considered as a mediocre fit. Values > .10 are not acceptable. The SRMR should be less than .05 for a good fit [32], whereas values smaller than .10 are still deemed acceptable. The comparative fit index (CFI) and the goodness of fit index (GFI) were also used to test how well the model fits the data. A CFI or GFI value over .90 generally indicates a reasonable fit between the model and the data but Hu and Bentler [32] recommend the use of a more severe criterion (≥.95) to describe a good fit. Correlations between BSL-23 and other psychological scales (BDI-II, BHS, BIS-10 and STAXI) were analysed, using the Bonferroni correction for multiple correlations. Correlation for ordinal data (Spearman’s rho) was performed to assess the association between BSL-23 and number of BPD criteria in the SCID-II [8]. Discriminant validity with a group of patients suffering from ADHD and no co-occurring BPD was also tested.

Finally, in order to assess BSL-23 sensitivity to change through therapy, scores before and after a four-week I-DBT [19, 20] were compared. The change of the BSL-23 scores before and after I-DBT was evaluated by paired-sample t-tests and Cohen’s d effect size.

## Results

### Demographic data

The clinical characteristics of the sample of 265 BPD patients (239 women, 90.8 % and 26 men, 9.8 %) are shown in Table 1. The median number of positive criteria in the SCID-II was 7 (min = 5; max = 9). Ages ranged from 18 to 58, with a mean of 32 years old (SD = 8.9). The mean number of years of education was 14 (SD = 2.5). Subjects were predominantly single (*N =* 186; 70.2 %) and without children (*N =* 194; 73.2 %). The educational level can be described as low (9–11 years) for 19.2 % (*N =* 51), medium (12–14 years) for 38.5 % (*N =* 102), and high (≥15 years) for 42.3 % (*N =* 112) of the sample. More than half of the sample (*N =* 149; 56.2 %) were neither studying nor working at the time of the study. Since no gender differences were found for all measures, the analyses were computed for the entire sample (men and women).

### Psychometric properties of the French BSL-23

#### Reliability

The original BSL-23 [15, 16] was tested on different samples of BPD patients and showed very good internal consistency (*N =* 379; Cronbach’s alpha = .97; *N =* 147; Cronbach’s alpha = .94; *N =* 35; Cronbach’s alpha = .96). In our sample (*N =* 265), the global Cronbach’s alpha was .94, and with the split-half method the reliability coefficient was .93. In the item-by-item reliability analysis, Cronbach’s alpha coefficients ranged from .936 to .942. Results indicate that the 23-item scale has high internal consistency. The BSL-23 mean score of the sample (*N =* 265) was 1.90 (SD = .88; min = .22; max = 3.83).

To study the test-retest reliability of the French BSL-23, a sub-sample of 61 BPD patients were asked to complete the instrument again after one week. This revealed a high correlation (*r =* .841; *p <* .001) between the first (m = 1.89; SD = 1.00) and second time (m = 1.73; SD = .91) the scale was completed, which suggests high test-retest reliability. In the original study, Bohus et al. [16] already found high test-retest reliability (*r =* .82; *p <* .001) in a sample of 35 subjects.

#### Factor structure

**Fig. 1:**
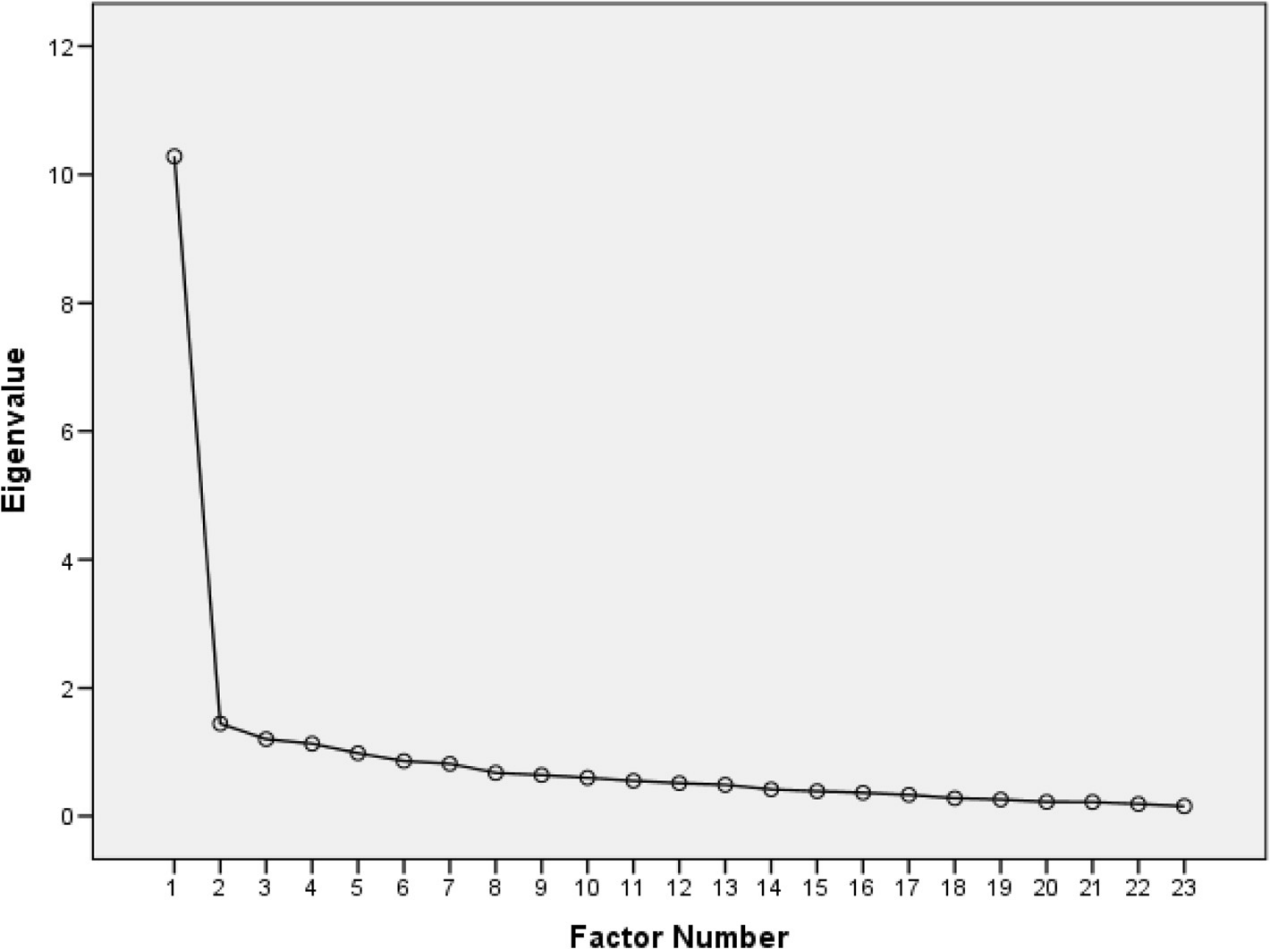
Scree plot of French BSL-23

The KMO measure of our data’s sampling adequacy was very high (.936) and Bartlett’s test of sphericity (3263.2) was highly significant (*p <* .001). Both measures indicated that the factor analysis is appropriate for our data. The factor analysis of the original BSL-23 [15, 16] suggested a one-factor structure, and both the principal component analysis and the scree plot of eigenvalues supported the dominance of a single factor, accounting for 40.6 % of the total variance. In our data, the single factor explained 44.7 % of variance. Although the EFA showed four factors with eigenvalues greater than 1.0 (10.285, 1.445, 1.201 and 1.131), cumulatively accounting for 61.1 % of the variance, the scree plot (Fig. 1) indicated a one-factor solution. Retaining all factors with eigenvalues greater than 1.0 is often an overestimation of the number of factors to be retained and Floyd and Widaman [34] suggest that the scree plot is a more useful guide. Another factor analysis was therefore conducted, specifying that a single factor should be identified. When one factor was fixed, all items showed factorial loadings equal or superior to .40, which is an acceptable level for a central factor (Table 2).

**Table 2:** Factor Structure of the French BSL-23

BSL items		Factor 1
BSL-1	It was hard for me to concentrate - *Il m’était difficile de me concentrer*	.483
BSL-2	I felt helpless - *J’étais désespéré(e)*	.787
BSL-3	I was absent-minded and unable to remember what I was actually doing - *J’avais l’esprit ailleurs et j’étais incapable de me rappeler ce que j’étais en train de faire*	.479
BSL-4	I felt disgust - *Je me suis senti(e) dégouté(e)*	.691
BSL-5	I thought of hurting myself - *J’ai pensé à me faire du mal*	.727
BSL-6	I didn’t trust other people - *Je n’avais pas confiance aux autres*	.399
BSL-7	I didn’t believe in my right to live - *Je ne croyais pas en mon droit de vivre*	.763
BSL-8	I was lonely - *J’étais seul(e)*	.425
BSL-9	I experienced stressful inner tension - *J’ai vécu une tension interne stressante*	.640
BSL-10	I had images that I was very much afraid of - *J’avais des images qui me faisaient peur*	.598
BSL-11	I hated myself - *Je me détestais*	.799
BSL-12	I wanted to punish myself - *Je voulais me punir*	.797
BSL-13	I suffered from shame - *J’ai éprouvé de la honte*	.682
BSL-14	My mood rapidly cycled in terms of anxiety, anger, and depression - *Mon humeur changeait rapidement passant de l’anxiété, à la colère et à la tristesse*	.682
BSL-15	I suffered from voices and noises from inside and/or outside my head - *J’ai entendu des voix et des bruits provenant de l’intérieur ou de l’extérieur de ma tête*	.399
BSL-16	Criticism had a devastating effect on me - *Les critiques d’autrui ont eu un effet dévastateur sur moi*	.703
BSL-17	I felt vulnerable - *Je me suis senti(e) vulnérable*	.689
BSL-18	The idea of death had a certain fascination for me - L’idée de la mort m’a fasciné(e)	.631
BSL-19	Everything seemed senseless to me - *Tout me paraissait vide de sens*	.727
BSL-20	I was afraid of losing control - *J’avais peur de perdre le contrôle*	.691
BSL-21	I felt disgusted by myself - *Je me suis senti(e) dégoûté(e) de moi-même*	.823
BSL-22	I felt as if I was far away from myself - *Je me suis senti(e) comme très éloigné(e) de moi-même*	.700
BSL-23	I felt worthless - *Je me suis senti(e) sans valeur*	.778

The goodness of fit test was good (chi square = 765.25, df = 230, *p <* .001), but results of the CFA with the one-factor and recommended fit indexes were less than satisfactory. The values of RMSEA (.114) and RMSR (.116) were above .10, which is usually considered to be unacceptable. The CFI (.82) and the GFI (.78) were inferior to .90 indicating a poor fit. Models with 2, 3 or 4 factors were examined but they didn’t provide for a better fit. The inadequacy of fit of our basic CFA model could be explained by the fact that several items, namely items 5, 7, 11, 12, 21, and 23, were highly inter-correlated (≥.70) (Table 3).

**Table 3:** Results of the Pearson’s r correlation coefficient for BSL-23 items^a^

	1	2	3	4	5	6	7	8	9	10	11	12	13	14	15	16	17	18	19	20	21	22	23
1	1.00																						
2	0.39	1.00																					
3	0.48	0.35	1.00																				
4	0.32	0.59	0.42	1.00																			
5	0.22	0.55	0.17	0.37	1.00																		
6	0.24	0.26	0.22	0.24	0.18	1.00																	
7	0.27	0.59	0.30	0.48	0.69	0.35	1.00																
8	0.11	0.34	0.09	0.27	0.29	0.23	0.26	1.00															
9	0.24	0.49	0.27	0.35	0.38	0.31	0.39	0.36	1.00														
10	0.26	0.42	0.32	0.41	0.37	0.20	0.42	0.25	0.39	1.00													
11	0.32	0.60	0.27	0.57	0.64	0.25	0.63	0.32	0.43	0.33	1.00												
12	0.31	0.57	0.29	0.48	**0.72**	0.23	**0.70**	0.24	0.37	0.42	0.68	1.00											
13	0.28	0.43	0.28	0.45	0.43	0.23	0.46	0.18	0.40	0.41	0.59	0.60	1.00										
14	0.34	0.54	0.35	0.45	0.41	0.28	0.41	0.29	0.45	0.39	0.47	0.45	0.48	1.00									
15	0.15	0.21	0.25	0.31	0.17	0.10	0.25	0.14	0.18	0.35	0.20	0.28	0.13	0.19	1.00								
16	0.36	0.49	0.38	0.49	0.36	0.28	0.49	0.29	0.44	0.39	0.47	0.44	0.46	0.55	0.28	1.00							
17	0.27	0.49	0.26	0.38	0.46	0.32	0.47	0.31	0.41	0.45	0.44	0.43	0.39	0.48	0.18	0.54	1.00						
18	0.14	0.42	0.17	0.35	0.59	0.17	0.61	0.30	0.35	0.36	0.44	0.53	0.32	0.32	0.37	0.34	0.37	1.00					
19	0.22	0.51	0.30	0.46	0.50	0.22	0.52	0.41	0.49	0.35	0.53	0.50	0.41	0.52	0.31	0.50	0.49	0.48	1.00				
20	0.27	0.49	0.32	0.41	0.42	0.30	0.41	0.25	0.50	0.47	0.51	0.49	0.50	0.50	0.21	0.45	0.55	0.31	0.46	1.00			
21	0.35	0.60	0.36	0.61	0.58	0.23	0.57	0.27	0.40	0.38	**0.76**	**0.71**	0.63	0.48	0.30	0.54	0.50	0.45	0.54	0.56	1.00		
22	0.26	0.47	0.41	0.46	0.36	0.28	0.41	0.24	0.43	0.42	0.51	0.50	0.44	0.45	0.37	0.48	0.51	0.36	0.55	0.60	0.58	1.00	
23	0.42	0.66	0.31	0.51	0.49	0.29	0.57	0.28	0.39	0.29	0.67	0.58	0.49	0.41	0.24	0.51	0.56	0.38	0.52	0.44	**0.72**	0.58	1.00

^a^Items with a level of correlation equal or above .70 are bold

#### Convergent validity

The following scales were used to analyse the convergent validity of the French BSL-23: BDI-II, BHS, BIS-10 and STAXI. All correlations are reported in Table 4. Because of the large number of correlations calculated from this sample, we applied the Bonferroni correction and focused on correlations that were significant at *p* ≤ .005. As found in previous reports [15, 16], the French BSL-23 score was highly correlated with depression severity, as measured by the BDI-II (*r =* .550), and with hopelessness, as measured by the BHS (*r =* .350). In addition, high scores on the BSL-23 were associated with state-anger (*r =* .482), trait-anger (*r =* .285), anger-in (*r =* .284) and anger-out (*r =* .194) subscales of the STAXI. Correlations between BSL-23 and motor impulsivity (sub-score of the BSI; *r =* .281) were also found. A positive correlation (*r*_*s*_ = .200, *n =* 265, p = .001) was found between number of positive criteria at the SCID-II and severity of symptoms in BSL-23.

**Table 4:** Correlations between French BSL-23 and other dimensions

scale scores	N^a^	BSL-23
BDI-II	245	.550*
BHS	245	.350*
BIS-10—Motor impulsivity	190	.281*
BIS-10—Cognitive impulsivity	190	.196
BIS-10—Non-planning impulsivity	190	.063
BIS-10—Total	190	.241*
STAXI—State Anger	220	.482*
STAXI—Trait Anger	220	.285*
STAXI—Anger-in	220	.284*
STAXI—Anger-out	220	.194*
STAXI—Anger Control	220	.341*

^a^N vary because of missing data

*Correlation significant at *p* ≤ .005

*BDI-II* Beck Depression Inventory II, *BHS* Beck Hopelessness Scale, *BIS-10* Barrat Impulsivity Scale, *STAXI* State-Trait Anger Expression Inventory

#### Discriminant validity

To determine whether the French BSL-23 discriminates BPD patients from other patient groups, the questionnaire was given to 45 patients (18 women, 40 % and 27 men, 60 %) with ADHD and no comorbid BPD. The demographic characteristics of the two groups showed no statistically significant differences. Independent sample tests (t = 8.084, *p <* .001) showed that BPD patients had higher BSL-23 scores compared with ADHD patients (m = .78; SD = .49). This result support the fact that the items of the BSL-23 were selected because of their ability to discriminate between BPD patients and patients with different axis I diagnoses [15, 16].

#### Sensitivity to change

We examined changes in the French BSL-23 scores in a sample of 92 BPD patients (87 women and 5 men) who participated in a four-week I-DBT. Patients completed the BSL-23 before and after the four-week program. BSL-23 scores decreased significantly after I-DBT (before: 2.00; SD = .91; after: 1.53; SD = .86) with a medium effect size (d = .53).

## Discussion

The aim of this study was to investigate the psychometric properties of a French version of the BSL-23 in a BPD sample. Our results showed that its psychometric properties were similar to those of the original [15, 16] and the Spanish [18] versions. The French BSL-23 had a high internal consistency and test-retest reliability and the factor analysis showed one highly dominant factor. The French version of the BSL-23 was correlated with depression (BDI-II), hopelessness (BHS), experience and expression of anger (STAXI), and impulsivity (BIS-10); the strongest correlation was found to be with depression severity (*r =* .550). Bohus et al. [16] already found moderate-to-high correlations between the BSL-23 and depression, as well as general severity of psychopathology and global well-being. This was supported in the study by Soler et al. [18], showing that the Spanish version of the BSL-23 positively correlated with measures of depression and anxiety symptoms, hostility, and impulsivity. The correlation with depression was also among the strongest ones in their study. BPD has a great comorbidity with depression [35] and there are similarities between depressive symptoms assessed by the BDI-II and BSL-23 items assessing suicidal ideations and dysphoria which are diagnosis criteria of BPD. The correlation between BSL-23 and hopelessness (BHS) was also expected as depressed BPD patients tend to exhibit high levels of hopelessness [36]. Associations with impulsivity (BIS-10) and anger (STAXI) measures show that the BSL-23 captures a wide range of BPD symptoms, including emotional and behavioral dysregulations. The positive correlation between severity of symptomatology measured by the BSL-23 and number of positive criteria at the SCID-II (BPD part) also supports this idea.

In our study, we also demonstrated the discriminant validity of the French BSL-23 when comparing BPD patients with ADHD patients. Although these two disorders are highly comorbid and share similar characteristics, such as impulsiveness and emotion dysregulation [37], the French BSL-23 was able to discriminate between the two conditions, a fact that demonstrates the specificity of the scale in assessing BPD symptomatology. This is consistent with the findings of Bohus et al. [16], showing the ability of the original version of the BSL-23 to distinguish between patients suffering from various psychiatric disorders, including ADHD, and patients suffering from BPD.

Besides being useful in assessing the disorder’s current severity, we found that the French version of the BSL-23 was sensitive to change after a four-week I-DBT intervention. Again, this is consistent with the results of previous studies showing that the original version of the BSL-23, as well as its Spanish version, had a good sensitivity to change either after 12 weeks of specific treatment for BPD or after a three-month DBT therapy, respectively [16, 18]. Further studies could investigate the relevance of this instrument to measure changes in borderline symptomatology after a longer psychotherapeutic intervention targeting BPD patients specifically, such as standard DBT [17] or Mentalization-Based Treatment [38].

Some limitations must be considered. First, the BSL-23 is a self-report measure and is obviously dependent on the introspective ability of the person. Nonetheless, the positive correlation between BSL-23 mean score and number of symptoms assessed by the SCID-II suggests that patients’ own evaluation was coherent with the clinician’s assessment of BPD. Another limitation should be reported with regard to the ADHD patients included in our study. They were clinically assessed to exclude a BPD diagnosis by expert psychiatrists, but they didn’t undergo the BPD interview of the SCID-II to confirm the absence of BPD.

## Conclusions

BPD is the most common personality disorder in clinical settings, but, to our knowledge, no self-report instruments in French was available to assess the severity of the disorder and its sensitivity to change following a therapeutic intervention. Our study showed that the French BSL-23 has good psychometric properties, provides a specific assessment of BPD symptomatology and is sensitive to change. This study provides a tool in French that is both easy and quick to use. It will allow clinicians and researchers to effectively measure borderline symptomatology.

## Abbreviations

ADHD, attention-deficit hyperactivity disorder; BDI-II, beck depression inventory II; BHS, beck hopelessness scale; BIS-10, barrat impulsivity scale; BPD, borderline personality disorder; BSL, borderline symptom list; BSL-23, borderline symptom list, short form;; CFA, confirmatory factor analysis; CFI, comparative fit index; DBT, dialectical behavior therapy; DIB-R, diagnostic interview for borderlines-revised; DIGS, diagnostic interview for genetic studies; DIVA, diagnostic interview for ADHD in adults; DSM-5, diagnostic and statistical manual of mental disorders, 5th edition; EFA, exploratory factorial analysis; GFI, goodness of ft index; I-DBT, intensive dialectical behavior therapy; KMO, kaiser-meyer-olkin; RMSEA, root mean square error of approximation; SCID-II, structured clinical interview for DSM-5 Axis II personality disorders; SPSS, statistical package for the social sciences; SRMR, standardized root mean square residua; STAXI, state-trait anger expression inventory; WURS, wender utah rating scale

## References

[1] Coid J, Yang M, Tyrer P, Roberts A, Ullrich S (2006). Prevalence and correlates of personality disorder in Great Britain. Br J Psychiatry.

[2] Grant BF, Chou SP, Goldstein RB (2008). Prevalence, Correlates, Disability, and Comorbidity of DSM-IV Borderline Personality Disorder: Results from the Wave 2 National Epidemiologic Survey on Alcohol and Related Conditions. J Clin Psychiatry.

[3] Trull TJ, Jahng S, Tomko RL, Wood PK, Sher KJ (2010). Revised NESARC personality disorder diagnoses: gender, prevalence, and comorbidity with substance dependence disorders. J Pers Disord.

[4] Skodol AE, Gunderson JG, McGlashan TH, Dyck IR, Stout RL, Bender DS, Grilo CM, Shea MT, Zanarini MC, Morey LC, Sanislow CA, Oldham JM (2002). Functional impairment in patients with schizotypal, borderline, avoidant, or obsessive-compulsive personality disorder. Am J Psychiatry.

[5] American Psychiatric Association (2001). Practice guideline for the treatment of patients with borderline personality disorder – Introduction. Am J Psychiatry.

[6] Kaess M, Brunner R, Chanen A (2014). Borderline personality disorder in adolescence. Pediatrics.

[7] American Psychiatric Association. Diagnostic and statistical manual of mental disorders. 5th ed. Washington: American Psychiatric Association; 2013.

[8] First M, Gibbon M, Spitzer R, Williams JBW, Smith Benjamin L (1994). Structured Clinical Interview for DSM-IV Personality Disorders (SCID-II).

[9] Zanarini MC (2003). Zanarini Rating Scale for Borderline Personality Disorder (ZAN-BPD): a continuous measure of DSM-IV borderline psychopathology. J Pers Disord.

[10] Arntz A, van den Hoorn M, Cornelis J, Verheul R, van den Bosch WMC, de Boer SF (2003). Reliability and validity of the borderline personality disorder severity index. J Personal Disord.

[11] Pfohl B, Blum N, St John D, McCormick B, Allen J, Black DW (2009). Reliability and Validity of the Borderline Evaluation of Severity Over Time (Best): A Self-Rated Scale to Measure Severity and Change in Persons With Borderline Personality Disorder. J Pers Disord.

[12] Bohus M, Limberger MF, Frank U, Sender I, Gratwohl T, Stieglitz RD (2001). Entwicklung der Borderline-Symptom-Liste. Psychother Psychosom Med Psychol.

[13] Bohus M, Limberger MF, Frank U, Chapman AL, Kuehler T, Stieglitz RD (2007). Psychometric properties of the Borderline Symptom List (BSL). Psychopathology.

[14] Zanarini MC, Gunderson JG, Frankenburg FR, Chauncey DL (1989). The revised diagnostic interview for borderlines: discriminating BPD from other axis II disorders. J Personal Disord.

[15] Wolf M, Limberger MF, Kleindienst N, Stieglitz R, Domsalla M, Philipsen A, Steil R, Bohus M (2009). Kurzversion der Borderline-Symptom-Liste (BSL-23): Entwicklung und Überprüfung der psychometrischen Eigenschaften. Psychother Psychosom Med Psychol.

[16] Bohus M, Kleindienst N, Limberger MF, Stieglitz RD, Domsalla M, Chapman AL, Steil R, Philipsen A, Wolf M (2009). The short version of the borderline symptom list (BSL-23): development and initial data on psychometric properties. Psychopathology.

[17] Linehan M (1993). Cognitive-Behavioral Treatment Of Borderline Personality Disorder.

[18] Soler J, Vega D, Feliu-Soler A, Trujols J, Soto A, Elices M, Ortiz C, Pérez V, Bohus M, Pascual JC (2013). Validation of the Spanish version of the borderline symptom list, short form (BSL-23). BMC Psychiatry.

[19] McQuillan A, Nicastro R, Guenot F, Girard M, Lissner C, Ferrero F (2005). Intensivedialectical behavior therapy for outpatients with borderline personality disorder who are in crisis. Psychiatr Serv.

[20] Perroud N, Uher R, Dieben K, Nicastro R, Huguelet P (2010). Predictors of Response and Drop-Out During Intensive Dialectical Behavior Therapy. J Pers Disord.

[21] Preisig M, Fenton BT, Matthey ML, Berney A, Ferrero F (1999). Diagnostic interview for genetic studies (DIGS): inter-rater and test-retest reliability of the French version. Eur Arch Psychiatry Clin Neurosci.

[22] Perroud N, Zewdie S, Stenz L, Adouan W, Bavamian S, Prada P, Nicastro R, Hasler R, Nallet A, Piguet C, Paoloni-Giacobino A, Aubry JM, Dayer A (2015). Methylation of serotonin receptor 3a in ADHD, borderline personality, and bipolar disorders : link with severity of the disorders and childhood maltreatment.

[23] Adult KJJS, ADHD. Diagnostic assessment and treatment, 3rd ed. London, England: Springer; 2012.

[24] Kessler RC, Adler L, Ames M, Delmer O, Faraone S, Hiripi E, Howes MJ, Jin R, Secnik K, Spencer T, Ustun TB, Walters EE (2005). The World Health Organization Adult ADHD Self-Report Scale (ASRS): A Short Screening Scale for Use in the General Population. Psychol Med.

[25] Romo L, Legauffre C, Mille S, Cheze N, Fougeres AL, Marquez S, Excoffier A, Dubertret C, Ades J (2010). Psychometric properties of the French version of the Wender Utah Rating Scale and Brown’s Attention Deficit Disorders Scale for adults. Encéphale.

[26] Ward MF, Wender PH, Reimherr FW (1993). The Wender Utah Rating Scale: an aid in the retrospective diagnosis of childhood attention deficit hyperactivity disorder. Am J Psychiatry.

[27] Fossati A, Novella L, Donati D, Donini M, Maffei C (2002). History of childhood attention deficit/hyperactivity disorder symptoms and borderline personality disorder: a controlled study. Compr Psychiatry.

[28] Beck AT, Steer RA, Ball R, Ranieri W (1996). Comparison of Beck Depression Inventories -IA and -II in psychiatric outpatients. J Pers Assess.

[29] Beck AT, Weissman A, Lester D, Trexler L (1974). The measurement of pessimism: the hopelessness scale. J Consult Clin Psychol.

[30] Bayle FJ, Bourdel MC, Caci H, Gorwood P, Chignon JM, Ades J, Loo H (2000). Factor analysis of french translation of the Barratt impulsivity scale (BIS-10). Can J Psychiatry.

[31] Spielberger C (1998). State-Trait Anger Expression Inventory, Research Edition. Professional Manual.

[32] Hu LT, Bentler PM (1999). Cutoff criteria for fit indexes in covariance structure analysis: Conventional criteria versus new alternatives. Structural Equation Modeling.

[33] Schermelleh-Engel K, Moosbrugger H, Muller H (2003). Evaluating the fit of structural equation models: Tests of significance and descriptive goodness-of-fit measures. Methods Psychological Res.

[34] Floyd FJ, Widaman KF (1995). Factor analysis in the development and refinement of clinical assessment instruments. Psychol Assess.

[35] Friborg O, Martinsen EW, Martinussen M, Kaiser S, Overgard KT, Rosenvinge JH (2014). Comorbidity of personality disorders in mood disorders: a meta-analytic review of 122 studies from 1988 to 2010. J Affect Disord.

[36] Soloff PH, Lynch KG, Kelly TM, Malone KM, Mann JJ (2000). Characteristics of suicide attempts of patients with major depressive episode and borderline personality disorder: A comparative study. Am J Psychiatry.

[37] Prada P, Hasler R, Baud P, Bednarz G, Ardu S, Krejci I, Nicastro R, Aubry JM, Perroud N (2014). Distinguishing borderline personality disorder from adult attention deficit/hyperactivity disorder: a clinical and dimensional perspective. Psychiatry Res.

[38] Bateman A, Fonagy P (2006). Mentalization-based treatment: a practical guide.

